# Modelling the Tumor Microenvironment: Recapitulating Nano- and Micro-Scale Properties that Regulate Tumor Progression

**DOI:** 10.3389/fcell.2022.908799

**Published:** 2022-06-14

**Authors:** Danielle Vahala, Yu Suk Choi

**Affiliations:** School of Human Sciences, The University of Western Australia, Perth, WA, Australia

**Keywords:** extracellular matrix, mechanobiology, invasion, hydrogel, stiffness, adhesion

## Abstract

Breast cancer remains a significant burden with 1 in 8 women affected and metastasis posing a significant challenge for patient survival. Disease progression involves remodeling of the extracellular matrix (ECM). In breast cancer, tissue stiffness increases owing to an increase in collagen production by recruited cancer-associated fibroblasts (CAFs). These stromal modifications are notable during primary tumor growth and have a dualistic action by creating a hard capsule to prevent penetration of anti-cancer therapies and forming a favorable environment for tumor progression. Remodeling of the tumor microenvironment immediately presented to cells can include changes in protein composition, concentration and structural arrangement and provides the first mechanical stimuli in the metastatic cascade. Not surprisingly, metastatic cancer cells possess the ability to mechanically adapt, and their adaptability ensures not only survival but successful invasion within altered environments. In the past decade, the importance of the microenvironment and its regulatory role in diseases have gained traction and this is evident in the shift from plastic culture to the development of novel biomaterials that mimic *in vivo* tissue. With these advances, elucidations can be made into how ECM remodeling and more specifically, altered cell-ECM adhesions, regulate tumor growth and cancer cell plasticity. Such enabling tools in mechanobiology will identify fundamental mechanisms in cancer progression that eventually help develop preventative and therapeutic treatment from a clinical perspective. This review will focus on current platforms engineered to mimic the micro and nano-properties of the tumor microenvironment and subsequent understanding of mechanically regulated pathways in cancer.

## 1 Introduction

As cancer, particularly metastasis, remains a leading cause of death globally (WHO, 2021), there is a call to action for researchers to develop novel approaches to enable new treatments. While the biochemical and genetic drivers of metastasis have been extensively studied, (Chatterjee et al., 2018; De Francesco et al., 2018; Siegel et al., 2018), there is a growing appreciation for the regulatory roles that biophysical cues play in tumor progression and the onset of metastasis. Changes to mechanical inputs such as stiffness (Sherman-Baust et al., 2003; Paszek and Weaver, 2004; Lu et al., 2011; Gierach et al., 2012) and ligand presentation (Allinen et al., 2004; Larsen et al., 2006; Oskarsson, 2013) are transduced to the cell nucleus via a process coined mechanotransduction. The microenvironment is composed of micro-and nanoscale features which undergo extensive remodeling during tumor development (Leiss et al., 2008; Wang et al., 2017). While these aberrant mechanical stimuli are acknowledged in tumor progression, the contribution and specific mechanism for this is still largely unknown. Understanding how mechanical stimuli regulate cancer cell fate largely relies on enabling biomimetic and tuneable materials that precisely recapitulate specific properties of the tumor microenvironment (TME) and more specifically the ECM. In this review, we discuss the functional consequences that alterations of the ECM, at the nano- and micro-scale, have on cancer cell growth and invasion and the platforms allowing the study of this complex disease.

## 2 What is the Tumor Microenvironment (TME)

**FIGURE 1:**
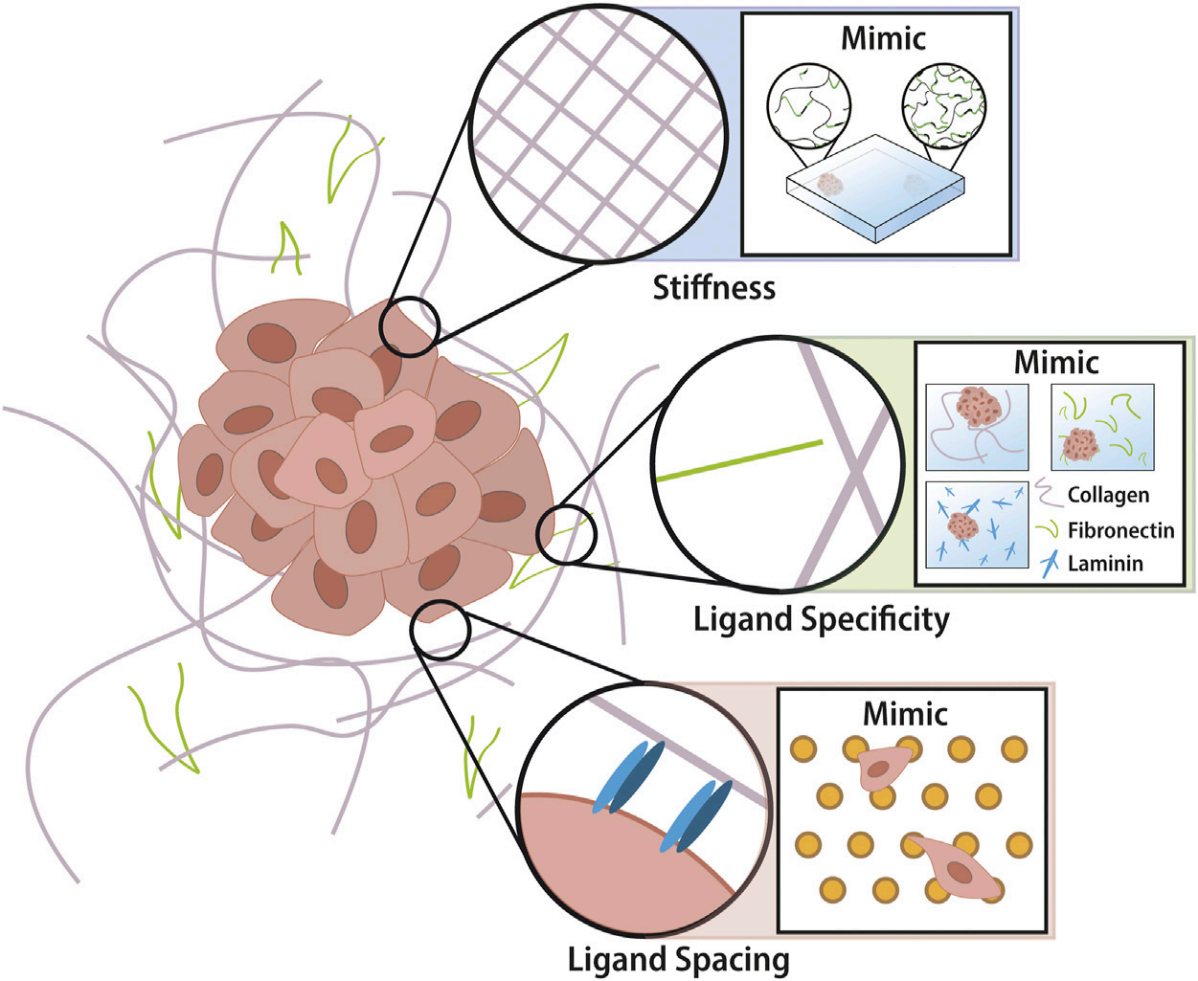
Tumor extracellular matrix in nano- and micro-scale. Properties of tumor ECM include microscale stiffness and ligand chemistry changes as well as nanoscale ligand spacing changes. The intelligent design of biomaterials allows recapitulation of these properties into tuneable devices for the study of cancer phenotypes. Hydrogels utilise cross-linking technology which investigators can control spatially across one gel by altering UV penetration. There is a multitude of biomaterials each utilising different backbones and employing different ligands for cells to adhere to (i.e. collagen = GFOGER and GelMA = RGD). Novel advancements in nanotechnology have enabled the production of a platform whereby the nano-spacing of ligands can be altered according to the micelle-nano-array of gold-nanoparticles that have attached peptides.

TME refers to the cellular (fibroblasts, immune cells, endothelial cells, and adipocytes, etc) and non-cellular (proteins that make up the ECM) components that form the microenvironment of a tumour. In this review, we focus on the non-cellular component, the ECM, present in all tissues and providing the physical scaffolding and biomechanical cues required for tissue morphogenesis, differentiation and homeostasis. The ECM consists of a multitude of proteins (e.g. collagen, laminin, and fibronectin) and glycosaminoglycans/proteoglycan (e.g. hyaluronic acid, heparin). Utilising certain cell membrane receptors (e.g., mainly integrins) cells can adhere to specific ligands expressed on the ECM. In this way, cells and the microenvironment maintain a dynamic dialogue, whereby healthy tissues remain in a state of homeostasis. This crosstalk is disrupted in tumorigenesis, where extensive remodification to the TME results in altered cell behaviour (Lu et al., 2011). The ECM is remodelled by ECM modifying factors (MMP and LOX) which alter cross-linking of the matrix or by paracrine signalling to cancer-associated fibroblasts (CAFs) resulting in increased deposition of ECM proteins (Özdemir et al., 2014; Rhim et al., 2014; Sahai et al., 2020; Lee and Chaudhuri, 2021). Changes in ECM composition by prolonged cross-linking/degradation will present altered mechanical properties (e.g., stiffness), cell-ECM ligand specificity (e.g., fibronectin dominant to collagen dominant), and altered spacing between ligands (e.g., nano-spacing of GFOGER, peptide in collagen that binds integrins α1β1 and α2β1) (Figure 1). As physical properties of the ECM influence cell behaviour via mechanotransduction and these properties are altered in cancer, acknowledging the cancer ECM and its regulatory role in controlling cell behaviour are critical for understanding cancer progression. (Young et al., 2016).

### 2.1 ECM Stiffness Activates Mechanotransduction Pathways to Regulate Cancer Fate

Changes to tissue stiffness following tumor development is one of the most well-established characteristics of the TME and this knowledge is exploited during cancer screening (e.g., palpation) (Khaled et al., 2004). Tumor stiffening is a downstream effect of increased deposition of ECM proteins (such as collagen) by the recruitment and activation of CAFs (Tlsty and Coussens, 2006). Collagen is increasingly more crosslinked by lysyl oxidase (LOX) which is implicated with increasing tension between cytoskeletal components and integrins and mediating cell-matrix focal adhesions (FA) (Choquet et al., 1997; Cox et al., 2013). Establishing a mechanical connection between cell and ECM, via FA, will result in the activation of various signalling cascades and the generation of intracellular force through actin-myosin. This process, coined mechanotransduction, begins at the cell-ECM interface whereby specific integrins bind to the ECM proteins to recruit and form actin stress fibers which maintain the cytoskeletal tension (Bershadsky et al., 2003). Filamentous Actin binds to Lamin-A within the nuclear membrane via adaptor proteins such as Nesprin and SUN (Wang et al., 2009), translating mechanical input to the nucleus of the cell. Tension applied to nuclear lamina (Lamin-A is one of the key building blocks) leads to translocation of mechanosensitive proteins such as transcriptional coactivator with PDZ-binding motif (TAZ) and yes-associated protein (YAP). This mechanotransduction pathway utilises biochemical and biomechanical signals to alter cancer cell growth, modes of invasion and eventually govern the fate of cells in the TME. Increased ECM stiffness enhances YAP and TAZ activity which evidence suggests can drive tumour-initiating cells (cancer stem cells (CSCs) to maintain self-renewal and tumor-initiation capacities of breast cancer cells and induce EMT (Cordenonsi et al., 2011; Dupont et al., 2011; Maugeri-Saccà et al., 2015). Alongside integrin-mediated mechanotransduction, increasing traction force not only regulates Rho-associated protein kinase (ROCK), a protein largely responsible for actin cytoskeleton dynamics and regulating breast cancer epithelial cell differentiation (Wozniak et al., 2003), but also activates mitogen-activated protein kinase (MAPK), which is responsible for many diverse cellular programs including cell proliferation, differentiation, motility and survival (Provenzano et al., 2009; Cargnello and Roux, 2011). Overall, a stiff ECM activates mechanotransduction which induces a gene expression signature in cancer cells that is associated with a greater risk for invasive breast carcinoma (Habel et al., 2004; Provenzano et al., 2009).

### 2.2 TME Stiffness is Dynamic at the Nanoscale

Previous investigations into the effects of stiffening on cancer phenotypes arose from the common consensus that tumors are stiffer than their healthy counterpart. However, the TME, and more specifically the ECM, has displayed levels of complexity not previously appreciated. A recent investigation into the nanoscale stiffness of breast explants revealed non-metastatic tumors with this characteristic increase in stiffness, but metastatic tumors with a “softer” more “heterogenous” stiffness profile with 3 distinct mechanical properties (Plodinec et al., 2012). These nanoscale measurements translate to clinical measurements, with high mammographic density associated with a greater risk of developing breast cancer, whilst low density is linked to an increased risk of cancer invasion (Ye et al., 2014). An examination of transgenic mice showed initial stiffening following tumor growth (spatially heterogeneous) followed by tissue softening at the onset of tumor dissemination as a result of ECM digestion (Sameni et al., 2000). These studies highlight the complexity of the TME and more importantly the dynamic nature of cancer ECM at the nanoscale.

### 2.3 Tumour Progression Involves Altered ECM Composition

#### 2.3.1 Protein Composition Changes and Integrin Specificity

In diseases where ECM is misregulated, changes to ECM composition results in changes to the ligand presentation (e.g., density (Sherman-Baust et al., 2003; Paszek and Weaver, 2004; Gierach et al., 2012) chemistry and geometry (Allinen et al., 2004; Larsen et al., 2006; Oskarsson, 2013)) which mediates integrin binding. Integrins are a family of transmembrane glycoproteins receptors consisting of 18 α and 8 β units which form heterodimers that mediate cell-matrix interactions (Ruoslahti, 1991; Hynes, 1992). As receptors, integrins mediate recognition of ECM constituents (e.g., cells that express α1β1 and α2β1 bind to GFOGER), recruiting focal adhesion complexes to establish traction force generation and actin filament assembly (Massia and Hubbell, 1991; Arnold et al., 2004). For this reason, integrin-mediated mechanotransduction has gained traction as a highly specific process that regulates cytoskeletal tension, intracellular signalling and gene expression related to proliferation, migration and survival. In breast cancer, an aberrant ECM can destabilise integrin-mediated adhesion resulting in enhanced metastatic potential and drug resistance (Young et al., 2020).

In the majority of cancers, significant accumulation of collagen has been associated with metastatic recurrence, aggressive behaviour, and chemoresistance (Lochter and Bissell, 1995; Ramaswamy et al., 2003; Oskarsson, 2013; Holle et al., 2016; Ondeck et al., 2019). Integrins α1β1 and α2β1 are known to be primarily collagen receptors and their expression has shown to play a role in melanoma cell migration in 3D collagen matrices (Koistinen and Heino, 2013). Increased expression of these two heterodimers is noted in melanoma metastatic cells when compared to cells in the primary tumor (Klein et al., 1991). Interestingly, studies utilising mammary epithelium and aorta-derived smooth muscles have shown collagen to activate distinct signalling pathways and alter cell behaviour independent of mechanical stimuli (stiffness) (Giancotti and Ruoslahti, 1999; Engler et al., 2004; Brownfield et al., 2013). Accompanying increased collagen deposition during a fibrotic response is the deposition of fibronectin. Fibronectin is a glycoprotein known to enhance the growth of breast epithelial cells (McIntosh et al., 2010), increase breast cancer invasion (via STAT3 and MAPK pathways) (Balanis et al., 2013) and is upregulated in circulating tumor cells (Raimondi et al., 2011). Cells adhere to RGD motifs expressed on fibronectin via the α5β1 receptor and αVβ3 which have emerged as essential mediators in many human carcinomas and are largely implicated in tumour proliferation and metastasis, with αVβ3 identified as a melanoma tumour progression marker (Hsu et al., 1998; Koistinen and Heino, 2013; Hou et al., 2020).

#### 2.3.2 Glycosaminoglycan Expression During Tumor Progression

Aside from ECM proteins, glycosaminoglycans (GAGs) have also proven essential in cancer progression. Hyaluronan is a GAG that is highly expressed in breast cancer (Karousou et al., 2014). Hyaluronan can interact with cancer cells via cell surface receptors CD44 and RHAMM (Toole, 2004). CD44 is a cell surface adhesion receptor (not an integrin) and is largely recognized as a cancer stem cell (CSC) marker expressed by almost every tumour cell (Jaggupilli and Elkord, 2012; Lin and Ding, 2017; Wang et al., 2018). Employing alternative signaling cascades then integrin-driven adhesion, CD44 has shown regulatory roles in the Hippo pathway (Wang et al., 2014) and interacts with RHAMM and ERK (Hamilton et al., 2007) as well as Rho-ROCK (Chellaiah et al., 2003; Ohata et al., 2012) to mediate breast cancer cell motility and enhance stemness of colon cancer-initiating cells. This offers an alternative perspective on the regulatory roles of GAGs and less understood cell-ECM interactions. Overall, these studies emphasize the specificity of cell-ECM adhesions which promote sustained altered signaling cascades and govern particular cell fates.

#### 2.3.3 Altered Nano-Spacing of Ligands in TME

Compositional remodeling due to increased deposition of ECM proteins, enhanced cross-linking by LOX and localized degradation by secreted MMP factors, has an implication in altering the spacing between individual ligands (e.g. GFOGER). Cells sense the variations in the spacing of ECM proteins through either single integrin proteins or recruitment of larger integrin-containing adhesion complexes (Oria et al., 2017). This spatial sensing has shown to play a role in physiological and pathological states (Daley et al., 2008). Oria et al., 2017 demonstrated that human breast myoepithelial integrin clustering and subsequent recruitment of focal adhesion is inhibited when cell-ECM interactions are separated by more than a few tens of nanometers. These nanometer-scale alterations have regulatory roles in cellular migration, morphology, focal adhesion assembly, cell adhesion and traction force generation (Arnold et al., 2004; Cavalcanti-Adam et al., 2006; Cavalcanti-Adam et al., 2007; Selhuber-Unkel et al., 2010; Oria et al., 2017). Interestingly, breast cancer cell survival, in response to chemotherapeutic treatment, is highly dependent on the nanoscale ligand spacing (Young et al., 2020).

Collagen fibres interact with each other at defined spacing intervals termed periodicity (Gelse et al., 2003; Young et al., 2016). This periodicity has shown intervals ranging from 63 to 72 nm and is the interval at which cells interact with the protein (Wallace et al., 2010; Young et al., 2016). *In vitro* studies have shown when periodicity is increased above 73 nm melanocytes and osteoclasts were inhibited in their ability to cluster integrins (Arnold et al., 2004). Fibronectin, also implicated in cancer, has shown a regular fibril arrangement of 42 nm in thick fibres and 84 nm in thin fibres. The fibrils at 84 nm will be unfolded and at a state of extension whilst the thick fibres are a result of staggered fibronectin dimers (Dzamba and Peters, 1991). It is hypothesized that the latter could be exacerbated in cancer due to the highly dense protein structures in the TME (Young et al., 2016). As the spatial organization of the available ligand binding partner changes, this mediates integrin clustering affecting force-mediated contractility of the cell and governing cell fate (Li et al., 2015). To confirm this theory, cartilage cells were placed on ligands of discreet spacing; the result was significantly greater cell area on the smaller nano-spaced ligands, inferring aberrant integrin-clustering on the larger nano-spacing (Li et al., 2015). In breast cancer cells, altering the nano-spacing of ligands showed altered cellular properties including morphology, focal adhesion formation, migration and chemoprotection (smaller ligand spacing hinders survival against chemotherapeutic drugs) (Young et al., 2020).

## 3 Tools for Recapitulating the Tumor Microenvironment

### 3.1 Stiffness Tunable Hydrogels

The TME offers a complex assortment of biochemical and biomechanical inputs that dynamically change following cancer progression and onset of metastasis. To better understand the functional consequences of ECM mechanics on cell phenotype, investigators have employed a combination of 2D and 3D hydrogel-based platforms. Being highly water-based and tunable, hydrogels offer superior recapitulation of specific characteristics of the ECM including stiffness, composition, and ligand spacing (Supplementary Table S1).

As mentioned previously, one of the most well-established characteristics of the TME is changes in tissue stiffness which is largely correlated with the risk of breast cancer development (Habel et al., 2004; Provenzano et al., 2008). Because of this, the production of biomaterials was largely centred around hydrogels whose chemistry would allow easy manipulation of stiffness. Synthetic gels such as polyacrylamide can produce discrete, static 2D gels of different stiffnesses and spatial gradients which offer greater biomimicry of the spatial heterogeneity seen *in vivo* (Engler et al., 2006; Insua-Rodríguez and Oskarsson, 2016; Hadden et al., 2017; Chin et al., 2021). Seeding metastatic breast cancer cells on uniform stiffness hydrogels which varied from 2.4–10.6 kPa demonstrated stiffness-dependent differences in traction forces, strain energies, and morphologies (Massalha and Weihs, 2017). As robust as PA is, the presence of free radicals during polymerization inhibits cell encapsulation and limits translatability from a 3D perspective.

Appreciation for the 3D architecture and its regulatory role on cell phenotype has geared the development of novel 3D-capable hydrogels. Collagen, being the primary constituent and major structural component in many tissues is very attractive for cell-ECM studies. Formed mainly from fibrous protein collagen type I, fabrication involves manipulating collagen concentration and raising the solution temperature until gelation occurs. Their soft mesh-like matrix makes collagen hydrogels ideal for invasion assays, with the most recent investigation showing collective cancer cell migration inhibited in the stiffest microenvironment (2.5 mg/ml) (Raghuraman et al., 2022). The drawback of this platform includes limited stiffness ranges (up to ∼5 kPa) and temporal tuneability, with reduced long-term stability and high batch-batch variability.

For these reasons, semi-synthetic materials such as gelatin methacryloyl (GelMA), synthesized from denatured collagen, has gained significant traction (Bertlein et al., 2017). Containing cross-linkable methacrylate groups, GelMA, will undergo polymerization upon the addition of a photoinitiator (Irgacure 2,959) and exposure to UV light. In this way, investigators gain greater tuneability over GelMA whilst still maintaining biological relevance (contains RGD). GelMA can also employ a gradient stiffness model which allows for a more holistic understanding of mechanically driven cell phenotypes (Major et al., 2019; Kim et al., 2020).

The use of these hydrogels allows for high throughput fabrication of readily adaptable and physiologically relevant environments, making it appealing for the study of ECM for cancer proliferation and invasion. Other approaches to mimicking the 3D microenvironment include 3D bioprinting which is beneficial in creating an exact replication of target tissue with consideration for cellular components, ECM and 3D spatial components (for reviews see; (Charbe et al., 2017; Ro et al., 2022)). Alternatively, the decellularization of tumors also offers a new Frontier in tissue engineering that maintains not only the mechanical components of the ECM but also the composition and structure (for review see; (García-Gareta et al., 2022)).

Stiffness is just one ECM characteristic supplying biophysical input to the cell. Tumor progression also proceeds extensive modification to ECM composition (i.e., going from fibronectin-laminin rich matrix to primarily collagen dominated) which alters the integrin profile and at the nanoscale alters the spacing at which cells are interacting with ECM proteins (Sherman-Baust et al., 2003; Allinen et al., 2004; Paszek and Weaver, 2004; Larsen et al., 2006; Gierach et al., 2012; Oskarsson, 2013).

### 3.2 Tools to Control ECM Composition

Polyacrylamide is inert in nature and requires conjugation with proteins to enable cell attachment. Using a bifunctional cross-linker sulfo-SANPAH, proteins can be covalently bound to the PA and enable cell attachment (Caliari and Burdick, 2016). Because of this, PA is an attractive choice when investigating the effects of specific adhesive ligands, as cell-ECM interactions can be tightly controlled (Tse and Engler, 2010). For 3D investigation different biomaterials which utilize different ligands can be employed. Collagen as mentioned previously expresses abundant GFOGER which will activate integrins α1β1 and α2β1. GelMA, adapted from native gelatin, contains RGD binding motifs (Bertlein et al., 2017) which will recruit integrins α5β1 and αVβ3. Alginate and polyethylene glycol diacrylate (PEGDA), do not express native ligands, similar to PA, and offers a “blank canvas” allowing preferential modification with adhesive ligands within a 3D context (Caliari and Burdick, 2016).

Hyaluronic acid (HA) as a hydrogel has several important advantages including its biological relevance and chemical tunability. HA functional groups can be modified to enable a wide range of cross-linking chemistries, useful for cellular mechanotransduction investigations (Fraser et al., 1997). Unlike collagen and GelMA, HA lacks integrin-mediated cell adhesion but presents cell surface markers including CD44 which can be beneficial when studying alternative cell-ECM adhesions and indirect pathways interacting with integrin-mediated mechanotransduction.

### 3.3 Tools for Investigating Ligand Nano-Spacing

Recapitulating and controlling for the nanoscale properties of the microenvironment remains a great challenge in biomaterials. Photolithography and optical lithography are the two most commonly used techniques in nanofabrication (Lohmüller et al., 2011). However, these two methods rely on light and achieving structural dimensions below 100 nm is hardly feasible (Lohmüller et al., 2011). Alternative methods, such as block copolymer micelle lithography (BCMN), rely on the self-organization of molecules to generate structural materials and can reach these sub-nanometer resolutions (Gates et al., 2004). BCMN involves the formation of spontaneous microphase-separated morphologies from amphiphilic block copolymers (Lohmüller et al., 2011). The distribution of nanoparticles due to block copolymer micelle self-assembly can be manipulated by changing micelle size, the concentration of polymer solution, the amount of metal precursor and the retraction speed from the substrate. Once the micro-arrays are established, peptides can be adhered to the nanoparticles enabling precise control over ligand spacing. Seeding cancer cells atop this platform has shown to influence key cellular properties such as morphology, focal adhesion formation, migration as well as drug sensitivity (Young et al., 2020).

## 4 Conclusion and Future Perspectives

The phenomenon responsible for the successful spread of primary cancer cells from their tumor microenvironment to secondary sites is still largely uncharacterized at the molecular level. Universally, the metastatic cascade employs mechanical challenges in which cancer cells will morphologically adapt to survive in these altered environments (Amos and Choi, 2021). Numerous *in vivo* and *in vitro* studies have examined the initial stages of tumor growth and invasion and begun to outline some of the nano- and micro-scale changes occurring in the TME–including changes to ECM stiffness, ligand chemistry and ligand nanospacing. Micro-scale changes, such as stiffness and ligand chemistry, have shown greater advancements in biomaterials than nano-scale properties where the primary obstacle has been developing tools *in vivo* to examine these changes and establishing platforms *in vitro* that allow for tuneability at this level. Overall, intelligent design of synthetic and biological biomaterials should incorporate these properties so that investigators can focus on the crucial differences between these properties and appreciate the role of the ECM and its regulation of cell fate even at the smallest, and perhaps most crucial, of metrics.
