# Supplementary material for: Modelling the Tumor Microenvironment: Recapitulating Nano- and Micro-Scale Properties that Regulate Tumor Progression
Source: Front Cell Dev Biol. 2022 Jun 14;10:908799. doi: 10.3389/fcell.2022.908799 (PMC9254080; doi:10.3389/fcell.2022.908799)
Supplement: Supplementary file 1 [file Table1.DOCX]

| **Biomaterial** | **Description** | **Ligand** | **Cell Receptor Interaction** | **Key Papers** |
| --- | --- | --- | --- | --- |
| **Alginate** | - Derived from brown algae - No native ligands, so requires modification - Most commonly uses ionic crosslinking (using divalent cations) but can utilise covalent crosslinking - Alginate can be easily dissolved for cell recovery making it desirable for cell-matrix investigations - Cannot be degraded naturally | ** Blank Slate | ** Dependent on choice of modification | (Lee and Mooney, 2012) |
| **Collagen** | - Normally sourced from rat tail tendon, bovine skin and tendon - Enzymatically degradable therefore favourable for invasion assays - Gelation at room temp produces low mechanical strength and limited long-term stability - Temperature largely effects the hydrogel architecture with lower temperatures producing longer fibrils | GFOGER | α2β1, α2β1 | (Shoulders and Raines, 2009) |
| **Polyacrylamide (PA)** | - Most widely used material and thus largely characterised - Wide range of mechanical stiffnesses - Requires protein conjugation for cell attachment - Only 2D capability - Fabrication occurs by reacting acrylamide monomer with bisacrylamide crosslinker – in which concentrations can be varied to achieve desired stiffness. | ** Blank Slate | ** Dependent on choice of ligand | (Pelham and Wang, 1997; Tse and Engler, 2010) |
| **Polyethylene glycol (PEG)** | - Allows high degree of user modification; PEG can be modified with functional groups that utilise chain-growth, step-growth, or mixed-mode polymerisation - Must be modified with adhesive ligand - Can be engineered to degrade via passive, proteolytic, or user directed modes - Chemically modified PEG can be used to crosslink other polymeric materials | ** Blank Slate | ** Dependent on choice of modification | (Lin and Anseth, 2009; Lutolf et al., 2003) |
| **Gelatin Methacryloyl (GelMA)** | - Synthesised from denatured collagen - Expresses native ligands for cell adhesion - Addition of photo crosslinker irgacure allows cross-linking of methacrylate sidechains by exposure to UV. - Offers greater mechanical tuneability and wider range of stiffnesses than collagen | RGD | α5β1, α8β1  αVβ1, αVβ3, αVβ5, αVβ6, αVβ8, αIIbβ3 | (Kim et al., 2020; Koistinen and Heino, 2013) |
| **Hyaluronic Acid** | - Sourced from bacterial fermentation or from animal products - Offers high degree of chemical modification enabling greater tuneability - Offers native interaction with cell receptors but does not support integrin-mediated cell adhesion | Repeating disaccharide unit of glucuronate and *N*-acetylglucosamine | CD44 | (Burdick and Prestwich, 2011; Dicker et al., 2014) |

Burdick, J.A., and G.D. Prestwich. 2011. Hyaluronic acid hydrogels for biomedical applications. *Advanced materials*. 23:H41-H56.

Dicker, K.T., L.A. Gurski, S. Pradhan-Bhatt, R.L. Witt, M.C. Farach-Carson, and X. Jia. 2014. Hyaluronan: a simple polysaccharide with diverse biological functions. *Acta biomaterialia*. 10:1558-1570.

Kim, C., J.L. Young, A.W. Holle, K. Jeong, L.G. Major, J.H. Jeong, Z.M. Aman, D.-W. Han, Y. Hwang, and J.P. Spatz. 2020. Stem Cell Mechanosensation on Gelatin Methacryloyl (GelMA) Stiffness Gradient Hydrogels. *Annals of biomedical engineering*. 48:893-902.

Koistinen, P., and J. Heino. 2013. Integrins in cancer cell invasion. *In* Madame Curie Bioscience Database [Internet]. Landes Bioscience.

Lee, K.Y., and D.J. Mooney. 2012. Alginate: properties and biomedical applications. *Progress in polymer science*. 37:106-126.

Lin, C.-C., and K.S. Anseth. 2009. PEG hydrogels for the controlled release of biomolecules in regenerative medicine. *Pharmaceutical research*. 26:631-643.

Lutolf, M.P., J.L. Lauer-Fields, H.G. Schmoekel, A.T. Metters, F.E. Weber, G.B. Fields, and J.A. Hubbell. 2003. Synthetic matrix metalloproteinase-sensitive hydrogels for the conduction of tissue regeneration: engineering cell-invasion characteristics. *Proceedings of the National Academy of Sciences*. 100:5413-5418.

Pelham, R.J., and Y.-l. Wang. 1997. Cell locomotion and focal adhesions are regulated by substrate flexibility. *Proceedings of the national academy of sciences*. 94:13661-13665.

Shoulders, M.D., and R.T. Raines. 2009. Collagen structure and stability. *Annual review of biochemistry*. 78:929-958.

Tse, J.R., and A.J. Engler. 2010. Preparation of hydrogel substrates with tunable mechanical properties. *Current protocols in cell biology*. 47:10.16. 11-10.16. 16.
